# Tannin alleviated reproductive dysfunction in pregnant ewes infected with *Haemonchus contortus*

**DOI:** 10.3389/fvets.2025.1531233

**Published:** 2025-02-11

**Authors:** Xin Li, Hai Xiang, Rong Liang, Xinyu Han, Rongzhen Zhong, Hongyu Liu, Yi Fang

**Affiliations:** ^1^Key Lab of Animal Production, Product Quality and Security, Ministry of Education, Jilin Agricultural University, Changchun, China; ^2^Key Laboratory of Livestock and Poultry Resource Evaluation and Utilization, Ministry of Agriculture and Rural Affairs, Jilin Agricultural University, Changchun, China; ^3^Jilin Provincial Laboratory of Grassland Farming, State Key Laboratory of Black Soils Conservation and Utilization, Northeast Institute of Geography and Agroecology, Chinese Academy of Sciences, Changchun, China; ^4^College of Advanced Agricultural Sciences, University of Chinese Academy of Sciences, Beijing, China

**Keywords:** gastrointestinal nematodes, tannin, pregnant ewes, hormones, placenta, fetus

## Abstract

**Introduction:**

*Haemonchus contortus* (*H. contortus*) infection has a significant impact on the health of pregnant ewes and adversely affects fetal development, highlighting the critical need for a non-toxic feed additive as an alternative and sustainable control strategy. Tannin is a kind of polyphenol compound, which has certain antiparasitic. The objective of this study was to evaluate the impact of dietary tannin supplementation on fecal egg count (FEC), packed cell volume (PCV), complete blood count (CBC), hormone levels, inflammatory markers, placental inflammation, and fetal growth and development in pregnant ewes infected with *H. contortus*.

**Methods:**

Hulunbuir ewes were randomly divided into three groups: control group (CON), gastrointestinal nematode infection group (GIN), and tannin group, which was infected by *H. contortus* with tannin feeding therapy (TAN). After artificial insemination was completed, and the ewes were confirmed for pregnancy and infection; stools were collected for FEC, and blood samples were collected for PCV and CBC, hormonal, and inflammation levels. The mRNA levels of hypothalamic–pituitary–ovarian axis-related hormone receptors and placental tissue inflammation genes were detected by quantitative reverse transcription polymerase chain reaction (RT-qPCR). Finally, fetal weights were measured, and fetal ovarian tissue samples were taken for transcriptomic analysis.

**Results:**

The results showed that tannins increased the levels of gonadotropin-releasing hormone (GnRH), follicle-stimulating hormone (FSH), luteinizing hormone (LH), estrogen (E_2_), progesterone (P_4_), human chorionic gonadotropin (hCG), red blood cell (RBC) counts, packed cell volume (PCV), and mRNA levels of gonadotropic axis receptors in pregnant ewes infected with *H. contortus* (*p* < 0.05). In addition, tannin reduced fecal egg count (FEC), leukocyte counts, and mRNA levels of inflammatory markers (*p* < 0.05). In addition, fetal ovarian sequencing further showed that tannin may alleviate the delay in fetal growth and development induced by *H. contortus* infection (*p* < 0.05).

**Conclusion:**

In summary, tannins have anthelmintic effects, restore reproductive hormone levels in pregnant ewes, reduce inflammation levels, and alleviate fetal growth retardation caused by *H. contortus* infection. Therefore, tannin is a suitable potential alternative to antibiotics as a feed additive.

## 1 Introduction

Ruminants are susceptible to gastrointestinal nematodes (GINs), especially *Haemonchus contortus* (*H. contortus*) infection (1). *H. contortus* infects ruminants, resulting in a serious decline in the level of economic production in the livestock sector and has become one of the major obstacles to the development of sheep farming (2). *H. contortus* is a highly pathogenic and voracious blood-feeding nematode, a parasite that causes anemia, low productivity, and decreased appetite in sheep (3, 4). Although *Haemonchus contortus* (*H. contortus*) infection causes significant harm to ruminant production, its effects during pregnancy remain underexplored in research.

Reproductive hormones are known to exert an important role in growth and development, pregnancy, and other reproductive activities in animals. However, GINs can disrupt with the synthesis and secretion of reproductive hormones in female animals (5). For example, the levels of serum hormones, such as estrogen (E_2_), testosterone (T), and receptors, associate with their biosynthesis are reduced in parasite-infected animals (6). Similarly, it can also lead to decreased levels of follicle-stimulating hormone (FSH) and luteinizing hormone (LH) (7). At the same time, other studies have shown that GINs can use host hormones to promote their own growth. For example, some *in vitro* experiments have shown that adding 80 ng/mL growth hormone accelerates *H. contortus* growth (8).

In addition, GINs also interfere with the female animal inflammatory system. According to Gossner et al. (9) and Estrada-Reyes et al. (10), *Teladorsagia circumcincta* induces significant changes in Type 1 T helper cells (TH1), for example, interleukin-2 (IL-2) and interleukin-8 (IL-8), and Type 2 T helper cells (TH2), for example, interleukin-4 (IL-4), interleukin-5 (IL-5), interleukin-6 (IL-6), interleukin-13 (IL-13), and interleukin-10 (IL-10) cytokine production. In addition, *H. contortus* infection has been shown to increase mRNA levels of interleukin-3 (IL-3), IL-6, IL-10, and tumor necrosis factor-*α* (TNF-α) in goats (11). Another study reported that the parasite induces an inflammatory response that leads to the accumulation of large numbers of neutrophils (NEU), lymphocytes (LY), and eosinophils (EOS) (12). Most importantly, infection during pregnancy severely affects the fetus, resulting in fetal mortality, stillbirth, and reduced litter size (13). For example, parasitic infections can impair the development of the fetus by increasing the expression of TNF-*α* in the placenta (14). However, the effects of GINs on reproductive performance in ruminants are not sufficiently reported, so further research is needed and treatment plans for *H. contortus* infection in pregnant animals are warranted.

In general, GINs were treated with albendazole or mebendazole, but it can lead to severe environmental pollution and chemical residues in meat, with serious negative effects (15, 16). Moreover, due to GIN high reproductive capacity and high levels of genetic diversity, resistance has evolved in several species such as sheep, goats, cattle, and horses (17). With the full implementation of the ban on antibiotic drugs in China in 2020, more and more research was focusing on the use of plant extracts in the treatment of parasites (18). Therefore, this has encouraged the search for an alternative drug to control GINs. Polyphenols have immune, antioxidant, and anti-inflammatory properties.

Tannins, which are found in most tanned plants, are a polyphenolic compound that has a variety of biological functions and can bind to proteins and macromolecular substances (19). Some studies have found that tannin-rich plants reduced GIN infection in sheep and goats (20), reduced the establishment of third-stage nematode larvae (iL3), and reduced the worm fertility and egg output (21). In addition, tannins indirectly improve the host’s immunity against GIN infection by increasing the proportion of absorbable amino acids in the host’s small intestine and can also directly block the metabolism and growth of GINs by binding proteins, carbohydrates, and esters in GINs (22). However, it has not been proven whether tannin has any therapeutic effect on the reproductive performance of pregnant animals infected with *H. contortus*.

This study aimed to investigate the effects of tannins on anthelmintic efficacy, inflammation levels, reproductive hormone levels, gonadal axis receptor mRNA expression, and placental inflammation in pregnant ewes infected with *H. contortus* and clarify that *H. contortus* infection during pregnancy in ewes can affect fetal development.

## 2 Materials and methods

### 2.1 *H. contortus* larvae

*H. contortus* was kept in the Jilin Provincial Key Laboratory of Grassland Farming Laboratory, Institute of Geography and Agroecology, Chinese Academy of Sciences, Jilin, and maintained by serial passage in helminth-free sheep. Infective L3 larvae (iL3s) were obtained by incubating eggs from the feces of sheep for 14 days at 25–28°C, according to the method of Paveto et al. (23).

### 2.2 Animals, diets, and experimental design

Thirty Hulunbuir ewes were selected with an average age of 160 ± 10.5-days and a body weight (BW) of 35 ± 1.2 kg. All ewes were individually housed and dewormed to ensure that no GINs were present in all lambs before the artificial insemination (AI). To verify a zero-egg burden, a fecal egg count (FEC) was done with a McMaster’s egg counting plate, and the results were expressed as eggs per gram of feces (EPG). The ewes were dewormed using abamectin (0.2 mg/kg BW, Novartis Animal Health Co, LTD, Shanghai), levamisole (7.5 mg/kg BW, Novartis Animal Health Co, LTD, Shanghai) and albendazole (5 mg/kg BW, Novartis Animal Health Co, LTD, Shanghai). After deworming, all ewes were randomly divided into three groups: control (CON) group, uninfected with *H. contortus*, *H. contortus* infected (GIN) group, orally dosed with L3 *H. contortus* larvae fluid (~10,000) (day of infection = Day 0), and tannin treatment (TAN) group, infected with *H. contortus* and given 4% tannin dosed on total dry matter (DM) supplementation (day of infection = Day 47). The condensed tannins in this study were supplied by Hunan Tea Group Co., Ltd., Hunan, China, and extracted from green tea leaves (*Camellia sinensis* L.). The chemical composition of the final product was determined by high-pressure liquid chromatography (Model Waters 600, Waters Co., Milford, MA, USA), following the method described by Paveto et al. (23). The final product contains condensed tannins (69.82%), flavonoids (15.06%), and steroids (15.12%). The condensed tannin concentration was determined to be 4% (0.6982 × 0.06 = 0.041) tannin dry matter (DM).

**Figure 1:**
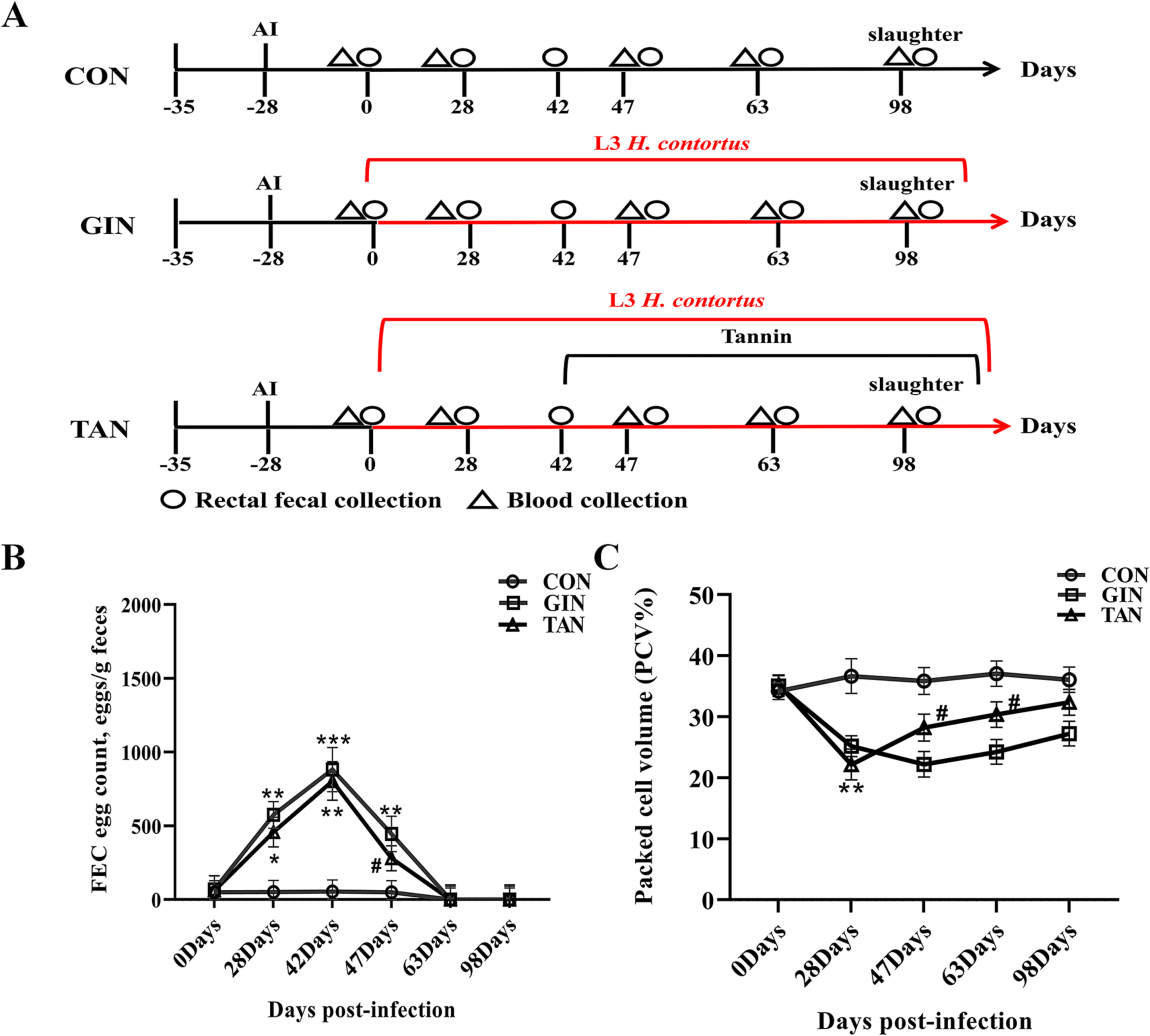
Effects of *H. contortus* and tannin on FEC, PCV, and CBC. **(A)** Experimental design of *H. contortus* infection in pregnant ewes and tannin treatment. **(B)** FEC of pregnant ewes infected with *H. contortus*. **(C)** PCV of pregnant ewes infected with *H. contortus*. Values were shown as means ± SEMs. *means *p* < 0.05; **means *p* < 0.01 vs. CON group. ^#^means *p* < 0.05 vs. GIN group.

Ram semen (Leke Biotechnology Co., Ltd., Inner Mongolia, China) was frozen in liquid nitrogen in 0.25 mL straws (Figure 1A, Day = −28) and then the frozen semen was removed from liquid nitrogen and placed in a 37°C water bath to thaw for 30s. Insemination was carried out using an insemination pipette according to the method of Pau et al. (24) to allow the semen to enter into the deep cervical cavity as far as possible. The insemination dose was 0.5 mL per ewe. After 28 days (Figure 1A, Day = 0), pregnancy diagnosis was conducted using a B ultrasonic instrument (XuZhou Electronic Equipment, China). Finally, three pregnant ewes with a high infection course, same litter size, and obvious pregnancy were selected in each group (B-ultrasonography and infection degree of other pregnant ewes were not significant and were not selected). After the pregnant ewes were infected with GIN, feeding trials were continued for 98 days (Figure 1A).

During post-infection rearing, each gestation was kept in a separate enclosure with free access to fresh water. All pregnant ewes were fed the same diet (Table 1) twice daily at 06:00 and 18:00. When the fecal worm egg count of pregnant ewes in the infected and tannin groups reached its peak, the tannin group diet was supplemented with given 4% of DM intake on 42 day of L3 infection (infected group and tannin treatment group), according to the method of Xiang et al. (25). Each feeding, according to the different feed intake of each pregnant ewe during the adaptation period, provides enough rations to ensure that the daily remaining feed of each pregnant ewe was the total feed.

**Table 1:** Ingredients and chemical composition of the basal experimental diet.

Items	Content
Ingredients	
Rape straw	20
Wheat straw	15
Soybean pod skin	25
Ground corn	20.35
Soybean meal	7.76
Wheat bran	7.00
Urea	1.50
NaCl	0.80
Limestone	0.49
Premix^a^	2.01
Total	100
Nutrient levels (*n* = 6)^b^	
DM	89.45
OM	91.23
CP	12.36
NDF	43.51
ADF	24.62
EE	3.25
ME/(MJ/kg)	10.38

CON and GIN, basal diet, TAN, basal diet plus 4% tannin DM ingredients.

a The premix provides the following ingredients per kilogram of diet: Ca 11.5 g, P 7.1 g, VA 30000 IU, VD 10000 IU, VE 120 mg, Na 3.5 g, Cu 12.5 mg, Fe 100 mg, Zn 80 mg, I 0.6 mg, Se 0.3 mg, Co 0.5 mg.

b ME are calculated values, and other nutrient levels are measured values.

### 2.3 Sampling and analytical procedures

On 0, 28, 42, 47, 63, and 98 days, first, 2 g crushed fecal samples of pregnant ewes were collected and mixed with 5 mL of water; then, saturated salt water was added to 28 mL and then filtered through a copper screen; the fecal fluid was injected into the McMaster’s egg count plate, and then left for 5 minutes to determine the FEC and expressed as EPG.

On 0, 28, 47, 63, and 98 days, pregnant ewes jugular vein blood samples were collected using vacuum aseptic sampling vessels without anticoagulant. Pregnant ewes jugular vein blood was extracted from different groups, and serum samples were collected and centrifuged at 1,000 rpm, 5 min, and 4°C, and the supernatant was preserved at −80°C. The concentrations of gonadotropin-releasing hormone (GnRH) (1.0 pg./mL, ml669855, Enzyme-linked Biotechnology Co., Ltd. Shanghai, China), LH (1.0 pg./mL, ml061712, Enzyme-linked Biotechnology Co., Ltd. Shanghai, China), FSH (1.0 mIU/mL, ml031713, Enzyme-linked Biotechnology Co., Ltd. Shanghai, China), E_2_ (1.0 pmol/L, ml490366, Enzyme-linked Biotechnology Co., Ltd. Shanghai, China), progesterone (P_4_) (1.0 pmol/L, ml036688, Enzyme-linked Biotechnology Co., Ltd. Shanghai, China), human chorionic gonadotropin (hCG) (0.1 mIU/mL, ml803396, Enzyme-linked Biotechnology Co., Ltd. Shanghai, China), IL-2 (0.75 pg./mL, JL22270-48 T, Jianglai Biotechnology Co., Ltd. Shanghai, China), IL-4 (0.1 pg./mL, CB10012-Sp, COIBO Co., Ltd. Shanghai, China), IL-10 (0.78–50 pg./mL, JHN99005, Jianglai Biotechnology Co., Ltd. Shanghai, China), and TNF-*α* (1 pg./mL, CB10066-Sp, COIBO Co., Ltd. Shanghai, China) were detected with ELISA kits, with optical density of the samples at 450 nm measured using a Thermomax microplate reader (BioTek Instruments, Winooski, VT, United States) following the manufacturer’s instructions. Blood samples from pregnant ewes on day 47 were shipped within 24 h to LABTARI (Beijing, China) for CBC. The PCV was determined by a hemocytometer (Sysmex XE-2100, Shanghai, China).

On 98 day, all pregnant ewes were fasted for 12 h and electrically stunned and slaughtered by exsanguination under commercial conditions. Hypothalamus, pituitary gland, ovary, placenta, and fetal ovary tissues were taken from pregnant ewes, placed in sterile 10 mL centrifuge tubes and labeled with serial numbers. All tissue samples were quickly snap-frozen in liquid nitrogen and stored at −80°C. To avoid the influence of different litter sizes on birth weight, ewes with the same multiple (two fetus) were selected, and the weight of the twins was weighed by an electronic scale.

### 2.4 RNA extraction and quantitative real-time polymerase chain reaction analysis

Hypothalamic, pituitary, ovarian, and placenta tissue samples were collected from three groups of pregnant ewes, and the tissues were stored in liquid nitrogen for follow-up tests. Total RNA from the hypothalamic, pituitary, ovarian, and placenta tissue RNA was treated with TRIzol (Takara, Tokyo, Japan). For cDNA synthesis, the Prime Script™ RT reagent kit (Takara, Tokyo, Japan) was used. The real-time PCR was performed in a Thermal SYBR^®^ Premix Ex Taq™ II (Takara, Tokyo, Japan) with specific primers designed (Table 2). Finally, 2^−∆∆Ct^ method was used to analyze the changes of hormone receptor levels and levels of indicators of inflammation between different groups.

**Table 2:** Primers used for qRT-PCR analysis.

Gene	Primer sequence (5′-3′)	Annealing temp. (°C)
*GnRH*	*F: CGATCAGCCAGTAGAACCTAAGTGC*	62
	*R: CCTCTGCCCAGTTTCCTCTTCAATC*	
*RFRP*	*F: CTGCTGCTGGAGATGCTGGATG*	62
	*R: GCTGGCTCTGATTCACGTCTTCC*	
*RFRPR*	*F: GTCAGGCTGGCATGGTTCTTGG*	62
	*R: CTCTGGCTTAGGGCTTGGCTTTC*	
*GnRHR*	*F: GCTGCCTCTTCATCATCCCTCTTC*	62
	*R: CCTCAGCCGAGCTTGTGGTATATTG*	
*FSH-R*	*F: CTTCAAGGAGCTGGTGTACGAGAC*	60
	*R: TTGCCGCAGTGACATTCAGTGG*	
*ESR-1*	*F: GACTGCTGCTGTGGCTGCTG*	60
	*R: TGCTGGTGGTGAAAGTGATACAGAC*	
*LH-R*	*F: ATCACACTGGAAAGATGGCACACC*	60
	*R: GAGGCAACATGGCGATGAGAGTAG*	
*PRLR*	*F: AGAACTTGTTCATCACACTCCACTG*	62
	*R: CAGGCAGAAGAGATCATCGGAATG*	
*IL-4*	*F: CAGCAGTGTTTGTGGTGAAGAATGG*	62
	*R: TGGTCAGGAAGGTGGCAGAGAAG*	
*IL-2*	*F: AACCCTTGTCTTGCATTGCACTAAC*	62
	*R: CTTTCATTGTGTTCCCCGTAGAGC*	
*TNF-α*	*F: AACGGCGTGGAGCTGAAAGAC*	62
	*R: CTGAAGAGGACCTGCGAGTAGATG*	
*IFN-γ*	*F: AGTTCTTGAACGGCAGCTCTGAG*	62
	*R: ATTTTGGCGACAGGTCATTCATCAC*	
*IL-10*	*F: CCGTGCTCTGTTGCCTGGTC*	62
	*R: GCTGGCTGGGAAGTGGGTAC*	
*TGF-β*	*F: GCGGAGCGACGAGGAATACTAC*	62
	*R: ACTGAGCCAGAGGGTGTTGTAAC*	
*β-actin*	*F: CGTGTTGGCGTAGAGGTCCTTG*	54–64
	*R: CCATCGGCAATGAGCGGTTCC*	

### 2.5 RNA extraction of fetus ovary tissue

Fetal ovarian tissue RNA was extracted from different groups, according to the manufacturer’s protocol (Magen), using TRIzol reagent. RNA quality was determined using the NanoDrop ND-2000 system (Thermo Scientific, United States). The integrity of the RNA values (RIN) by biological analyzer Agilent 4150 is used in the analysis system (Agilent Technologies, CA, United States).

#### 2.5.1 Library preparation and sequencing

Samples were processed following the manufacturer’s protocol for an ABclonal mRNA-seq Lib Prep Kit (ABclonal, China). This step was followed by first-strand cDNA synthesis using reverse transcriptase (RNase H). The second-strand cDNA was done using the synthesis of DNA polymerase I, RNAseH, buffer, and dNTPs. Second, the PCR products were purified by the AMPure XP system. The library size and mass were assessed by analysis in Agilent Bioanalyzer 4150 system. Finally, library preparations were sequenced using the reagents provided in the Illumina NovaSeq 6000.

#### 2.5.2 Data analysis and differential expression analysis

The raw data obtained by RNA-seq sequencing were used to remove low-quality reads and reads with N ratios greater than 5% using the Illumina platform. HISAT2 software^1^ was used to determine DEGs |log2FC| > 1 in the three groups and that (*p* ≤ 0.05) as significantly differentially expressed genes.

#### 2.5.3 Enrichment analysis of GO and KEGG

The GO function analysis of DEGs was conducted using cluster Profiler R software, with overrepresented functions filtered at a threshold of *p* ≤ 0.05. Finally, KEGG enrichment analysis identified significantly enriched pathways for DEGs under the threshold of *p* ≤ 0.05.

### 2.6 Statistical analysis

Three replications of each experiment were performed. Sigma Plot is used for statistical analyses, and Prism 5.0 was used for drafting. Student’s *t*-test was used for the statistical analysis of two groups. Statistical analyses of more than two groups were performed using analysis of variance (ANOVA). When *p* < 0.05, the statistics were significant.

## 3 Results

### 3.1 Effects of *H. contortus* and tannin on FEC, PCV, and CBC

It can be seen from Figure 1 that there were no significant differences in FEC and PCV among the three groups on day 0 (*p* > 0.05). As for FEC, from days 28 to 42, compared with the CON group, the FEC of the GIN and TAN groups increased significantly (Figure 1B, *p* < 0.05) and reached the peak on day 42. On day 47, compared with the GIN group, the FEC of the TAN group decreased significantly (Figure 1B, *p* < 0.05).

As for PCV, compared with the CON group, the PCV of the GIN and TAN groups decreased significantly on day 28 (Figure 1C, *p* < 0.05). On day 47, compared with the GIN group, the PCV of the TAN group increased significantly (Figure 1C, *p* < 0.05). On days 63–98, there were no significant differences in FEC and PCV among the three groups (Figures 1B,C).

The effects of tannins on CBC in pregnant ewes infected with *H. contortus* on day 47 were presented (Table 3). Compared to the CON group, LY%, MONO%, EOS%, NEU%, and PLT of the GIN group were significantly increased, and RBC was significantly decreased (*p* < 0.05). Furthermore, tannin supplementation was effective in restoring these blood parameters (*p* < 0.05). However, there were no significant changes in MCH, MCV, MCHC, RDW, MPV, PDW, and P-LCR% (*p* > 0.05).

**Table 3:** Routine blood test in pregnant ewes on day 47.

Complete blood count	Groups		
	CON	GIN	TAN
LY%	61.42 ± 1.21^a^	79.80 ± 1.35^b^	69.37 ± 1.17^a^
NEU%	28.82 ± 2.65^a^	44.58 ± 2.34^b^	35.86 ± 2.03^a^
EOS%	0.35 ± 0.02^a^	0.54 ± 0.04^b^	0.45 ± 0.06^a^
BAS%	0.24 ± 0.13	0.31 ± 0.15	0.27 ± 0.44
MONO%	5.62 ± 1.84^a^	7.58 ± 1.98^b^	6.22 ± 1.78a
RBC (10^12^/L)	11.9 ± 1.21^a^	8.0 ± 1.63^b^	9.6 ± 1.74^a^
MCH (pg)	34.95 ± 0.48	34.40 ± 0.70	35.89 ± 0.61
MCHC (g/dL)	386.96 ± 16.34	380.70 ± 17.60	385.73 ± 16.38
MCV (fL)	9.86 ± 0.15	9.52 ± 0.2	9.85 ± 0.17
RDW (CV%)	8.96 ± 0.02	8.95 ± 0.01	8.94 ± 0.05
PLT (10^9^/L)	240.56 ± 28.95^a^	311.51 ± 35.36^b^	254.45 ± 39.27^a^
MPV (fL)	9.58 ± 0.28	9.56 ± 0.35	9.85 ± 0.47
PDW (CV%)	8.96 ± 0.075	8.86 ± 0.06	8.93 ± 0.048
P-LCR%	13.59 ± 0.18	13.26 ± 0.25	13.25 ± 0.34

Means (±SD) of lymphocyte (LY%), eosinophil (EOS%), neutrophil% (NEU%), basophil% (BAS%), monocyte%(MONO%), red blood cell (RBC), mean corpuscular hemoglobin (MCH), mean corpuscular volume (MCV), mean corpuscular hemoglobin concentration (MCHC), red cell distribution width (RDW), blood platelet (PLT), mean platelet volume (MPV), platelet distribution width (PDW), and platelet-larger cell% (P-LCR%) in the among groups: infected with *H. contortus* and non-infected without tannins (GIN and TAN groups, respectively). a, b, c Different superscripts indicate significant differences among the three groups (*p* < 0.05).

### 3.2 Effects of *H. contortus* and tannin on inflammation levels

**Figure 2:**
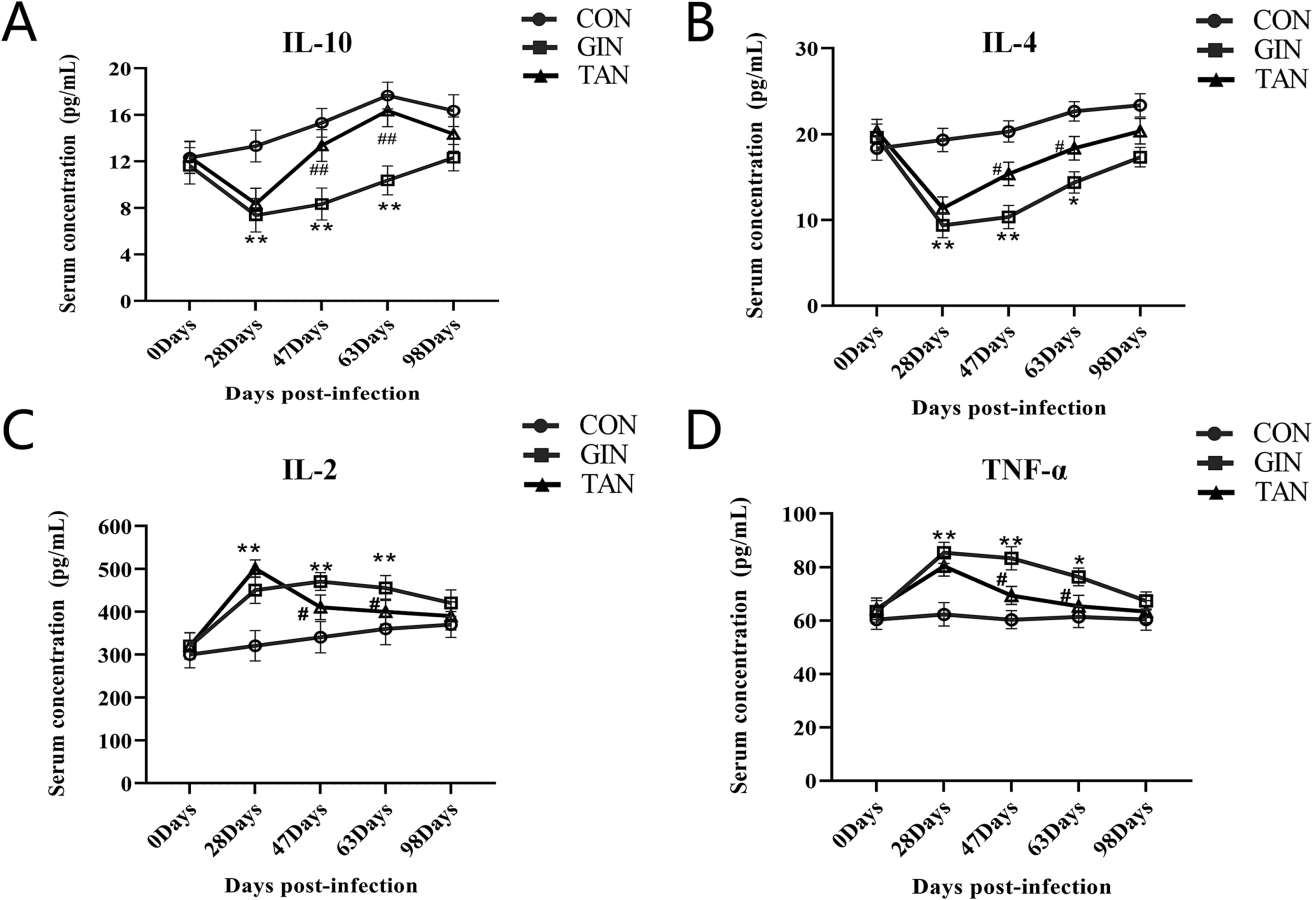
Effects of *H. contortus* and tannin on inflammation levels. **(A)** Effect on serum IL-10 concentration in pregnant ewes. **(B)** Effect on serum IL-4 concentration in pregnant ewes. **(C)** Effect on serum IL-2 concentration in pregnant ewes. **(D)** Effect on serum TNF-*α* concentration in pregnant ewes. *means *p* < 0.05; **means *p* < 0.01 vs. CON group. ^#^ means *p* < 0.05 vs. GIN group.

As shown in Figure 2, from days 0 to 28, IL-10 and IL-4 were significantly decreased, and IL-2 and TNF-*α* were significantly increased in the GIN and TAN groups compared to the CON group (Figures 2A–D, *p* < 0.05). From days 47 to 63, there was significant increase in IL-10 and IL-4 and decrease in IL-2 and TNF-α in the TAN group compared to the GIN group (Figures 2A–D, *p* < 0.05). In addition, on day 98, there were no significant differences among the three groups (Figures 2A–D, *p* > 0.05).

### 3.3 Effects of *H. contortus* and tannin on mRNA expressions of hormones and receptors in hypothalamus–pituitary–gonadal axis

**Figure 3:**
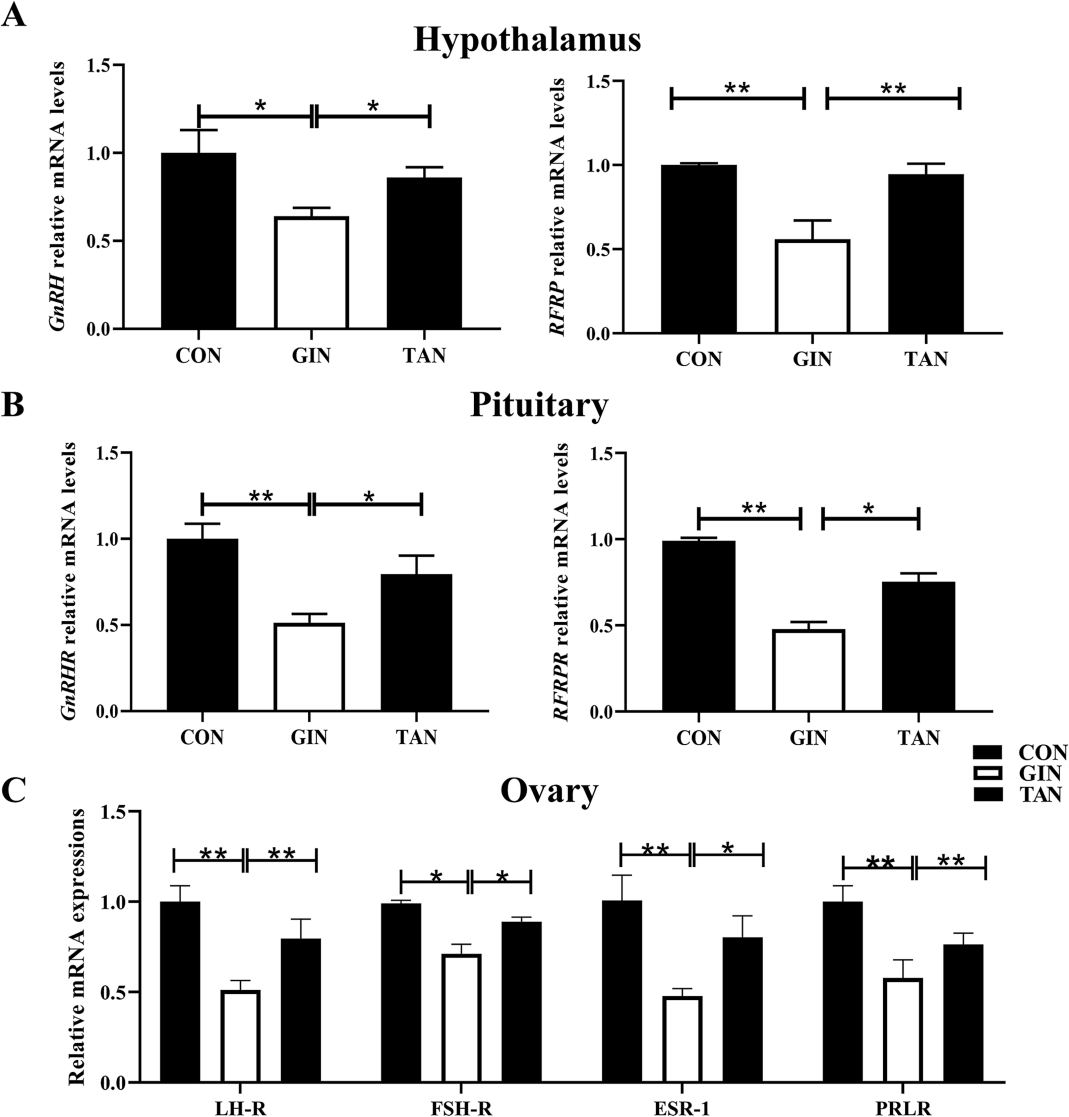
Effects of *H. contortus* and tannin on mRNA expressions of hormones and receptors in hypothalamus–pituitary–gonadal axis. **(A)** Relative expression levels of GnRH and RFRP in the hypothalamus of pregnant ewes. **(B)** Relative expression levels of GnRHR and RFRPR in the pituitary of pregnant ewes. **(C)** Relative expression levels of LH-R, FSH-R, ESR-1, and PRLR in the ovary of pregnant ewes. Values were shown as means ± SEMs. *means *p* < 0.05; **means *p* < 0.01.

The results showed that the mRNA levels of the GnRH and RFRP of the hypothalamic, GnRHR and RFRPR of the pituitary, and the FSH-R, LH-R, ESR-1, and PRLR of ovarian in the GIN group were significantly decreased than the CON group (Figures 3A–C, *p* < 0.05). The abnormal expressions of these genes were recovered in the TAN group (*p* < 0.05).

### 3.4 Effects of *H. contortus* and tannin on sex hormone levels

**Figure 4:**
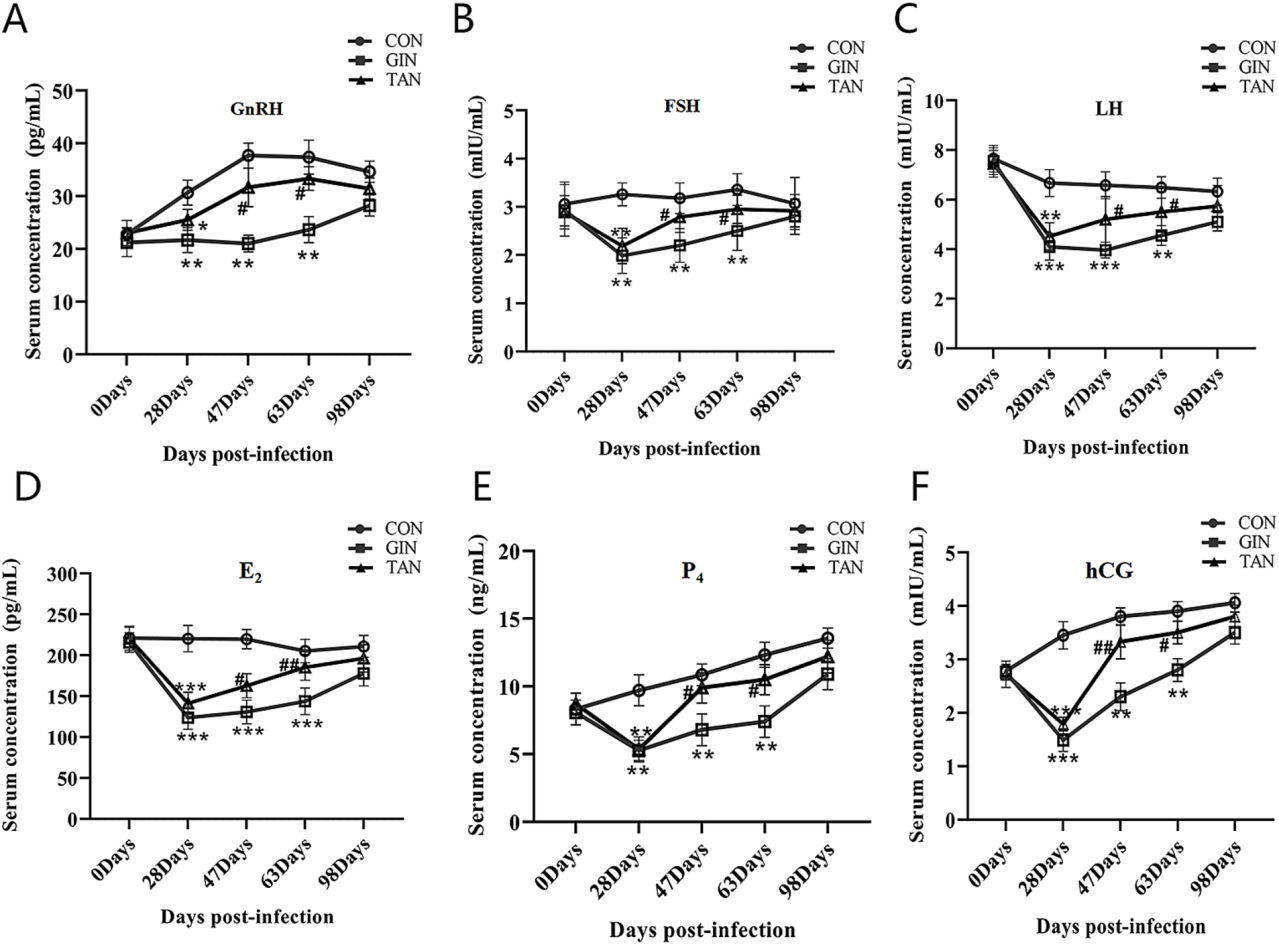
Effects of *H. contortus* and tannin on sex hormone levels. **(A)** Effect on serum GnRH concentrations in pregnant ewes. **(B)** Effect on serum FSH concentrations in pregnant ewes. **(C)** Effect on serum LH concentrations in pregnant ewes. **(D)** Effect on serum E_2_ concentrations in pregnant ewes. **(E)** Effect on serum P_4_ concentrations in pregnant ewes. **(F)** Effect on serum hCG concentrations in pregnant ewes. *means *p* < 0.05; **means *p* < 0.01 vs. CON group. #means *p* < 0.05 vs. GIN group.

To further investigate the effects of infection with GINs on the reproductive performance of pregnant ewes, we examined changes in hormone levels in pregnant ewes. As illustrated in Figure 4, on day 0, no significant differences in the levels of six hormones were observed among the three groups (Figures 4A–F, *p* > 0.05). From days 28 to 63, GnRH, FSH, LH, E_2_, P_4,_ and hCG levels in both the GIN and TAN groups were significantly lower than those in the CON group (Figures 4A–F, *p* < 0.05). From days 47 to 63, however, these hormone levels (GnRH, FSH, LH, E_2_, P_4_, and hCG) significantly increased in the TAN group than the GIN group (Figures 4A–F, *p* < 0.05).

### 3.5 Effects of *H. contortus* and tannin on mRNA expressions of placental inflammation indicators

**Figure 5:**
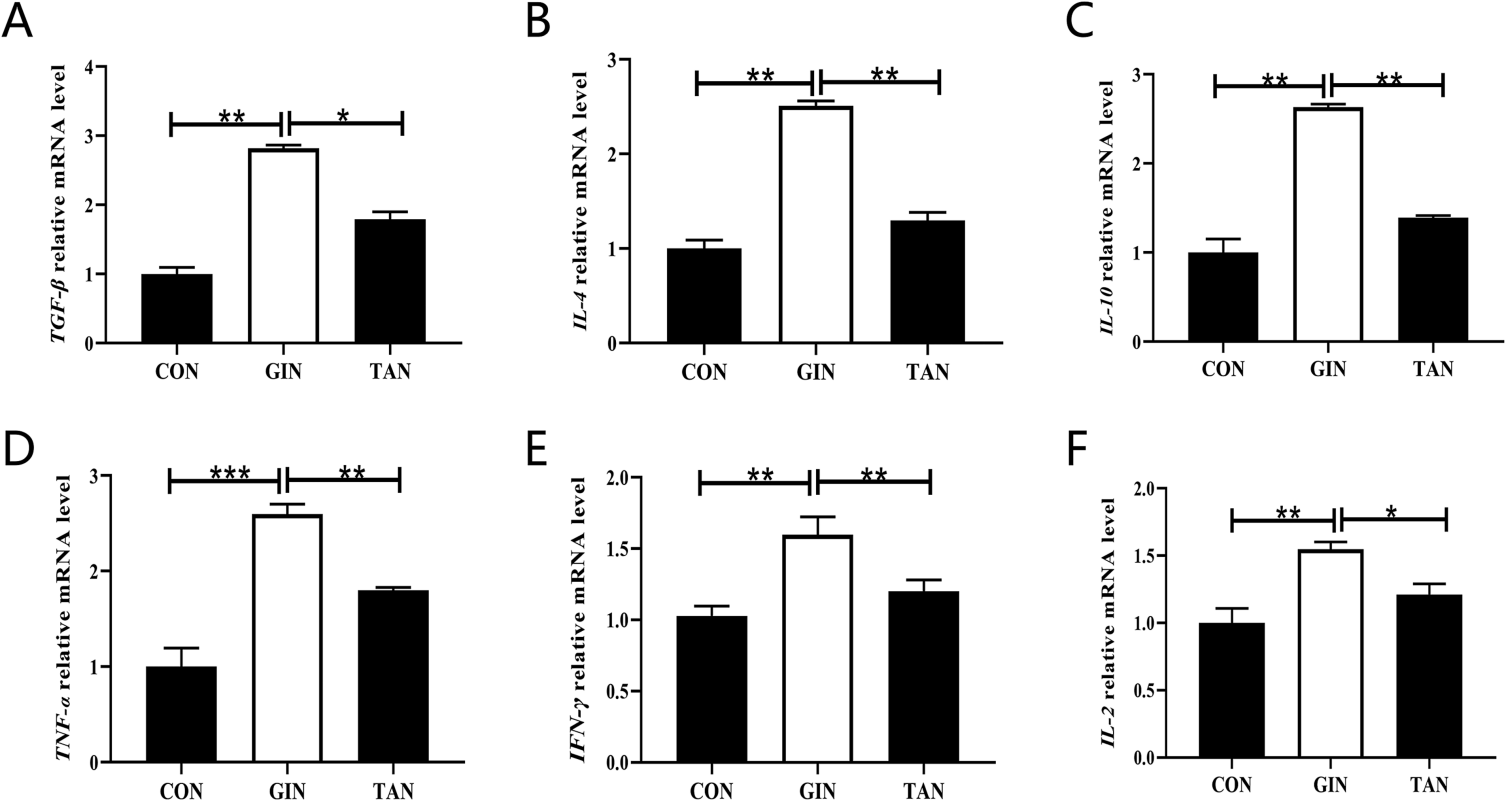
Effects of *H. contortus* and tannin on mRNA expressions of placental inflammation indicators. **(A–C)** The anti-inflammatory factor TGF-*β*, IL-4, and IL-10 mRNA levels. **(D–F)** The pro-inflammatory factor TNF-α, IFN-*γ*, and IL-2 mRNA levels. Values were shown as means ± SEMs. *means *p* < 0.05; **means *p* < 0.01.

It is hypothesized that the damage caused by *H. contortus* infection may impair fetal development through placental pathways. We further analyzed placental inflammatory responses. Compared with the CON group, the mRNA levels of anti-inflammatory cytokines TGF-*β*, IL-4, and IL-10 were significantly increased (Figures 5A–C, *p* < 0.05), as well as pro-inflammatory cytokines IL-2, TNF-*α*, and IFN-*γ* were significantly elevated in the GIN group (Figures 5D–F, *p* < 0.05). By contrary, in the TAN group, the levels of pro-inflammatory and anti-inflammatory factors were restored (Figures 5A–F, *p* < 0.05).

### 3.6 Effects of *H. contortus* and tannin on fetus weight and transcriptomic analysis of fetus ovary

**Figure 6:**
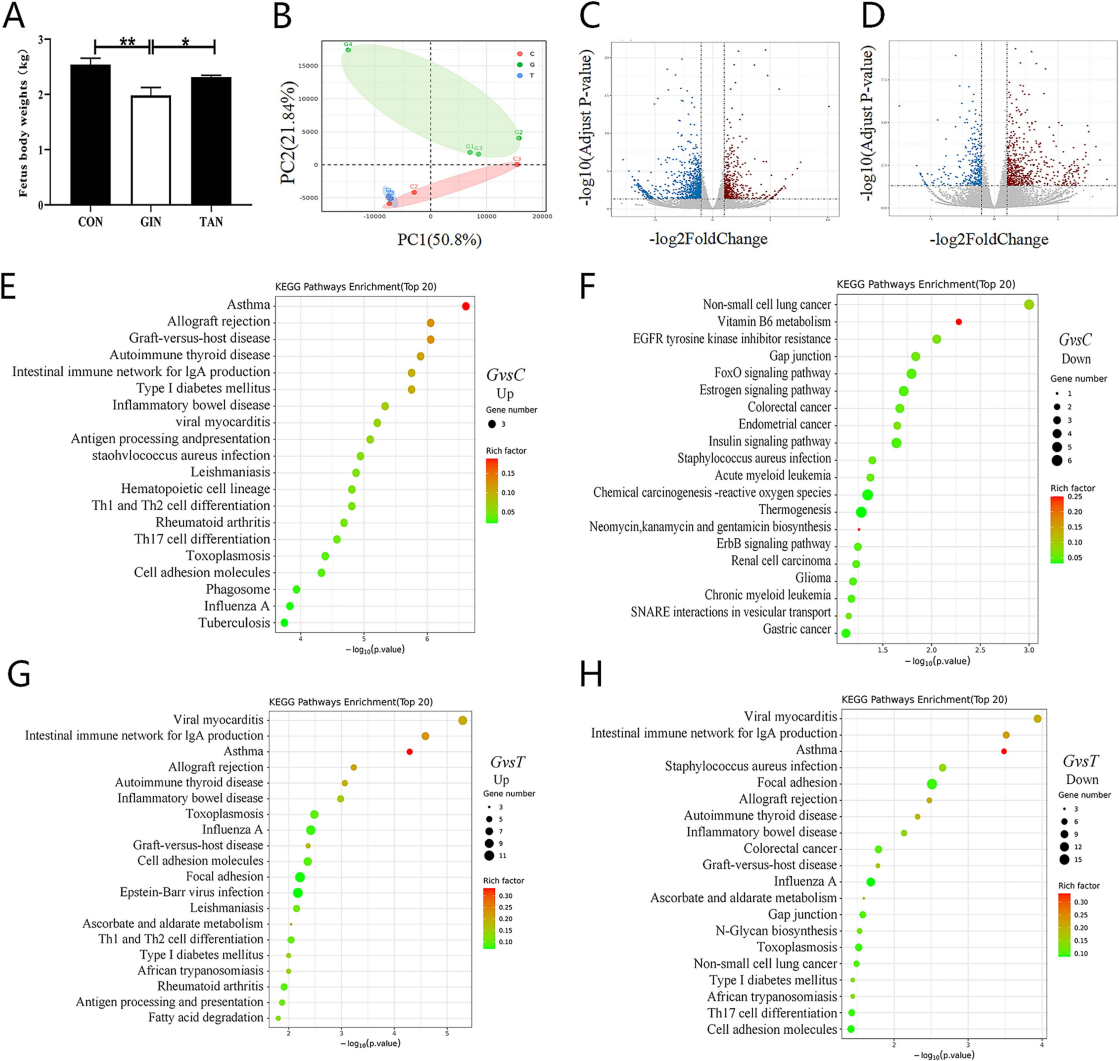
Effects of *H. contortus* and tannin on fetus body weight and transcriptomic analysis of fetus ovary. **(A)** Fetus weight infected with *H. contortus.* **(B)** PCA among CON (*n* = 3), GIN (*n* = 3), and TAN (*n* = 3). **(C,D)** Volcano plot of differentially expressed genes among the CON, GIN, and TAN groups (differential fold >1.5, *p* < 0.05). The horizontal axis represents log2 (fold change), and the vertical axis represents −log10 (*p*-value). **(E,F)** Top 20 of KEGG enrichment pathway of up- and downregulated genes between the CON and GIN groups. **(G,H)** Top 20 of KEGG enrichment pathway of up- and downregulated genes between the TAN and GIN groups. Pathways are sorted by −log10 (*p*-value) corresponding to each entry. Each dot in the figure represents a pathway; smaller *p*-values are represented by colors closer to red, while larger dots indicate a greater number of genes in the pathway.

Fetus weight in the GIN group was significantly reduced compared to the CON group (*p* < 0.05) but significantly increased after tannin supplementation (Figure 6A). To determine whether reproductive dysfunction induced by *H. contortus* was heritable and explore the potential ameliorative effects of tannin, the functions and biological changes in fetal ovaries were systematically analyzed through transcriptomics. Principal component analysis (PCA) revealed a clear separation between the three sample groups (Figure 6B). *H. contortus* alter the expression of 942 genes, with 636 upregulation and 306 downregulation, while tannins affected 1,464 genes, with 541 upregulation and 923 downregulation (Figures 6C,D, *p* < 0.05).

Based on Kyoto Encyclopedia of Genes and Genomes (KEGG) pathway enrichment analysis, compared with the CON group, the enriched pathways of the upregulated genes in the GIN group were mainly concentrated in the TH1 and TH2 cell differentiation, TH17 cell differentiation, and intestinal immune network for IgA production signaling pathway. Compared with the CON group,the enriched pathways of downregulated genes in the GIN group were mainly concentrated in Gap junction, FoxO signaling pathway, and Estrogen signaling pathway (Figures 6E,F, *p* < 0.05).

Compared with the GIN group, the enriched pathways of upregulated genes in the TAN group were mainly concentrated in the TH1 and TH2 cell differentiation, inflammatory bowel disease, and intestinal immune network for IgA production signaling pathways. The enriched pathways of the downregulated genes in the TAN group were mainly concentrated in Gap junction, TH17 cell differentiation, and intestinal immune network for IgA production signaling pathway (Figures 6G–H, *p* < 0.05).

## 4 Discussion

It is well known that tannin affects the reproductive performance of animals (26), but little is known about their therapeutic effect *H. contortus* infection in pregnant ewes. This study assessed the impact of tannin on the reproductive performance of pregnant ewes following *H. contortus* infection, focusing on its effects on deworming ability, reproductive hormone levels, gonadal axis receptor levels, inflammatory markers, and fetal development. We reported that tannin exhibited significant deworming effects and mitigated *H. contortus* infection’s impact on the growth and development of both pregnant ewes and their fetuses.

FEC, commonly used to indicate GIN infection in ruminants, is closely related to worm load and is the best phenotypic marker of GIN infection (27). The results of this study showed that tannin could reduce FEC in pregnant ewes, and this effect increased with the extension of feeding time. It is consistent with the results of previous reports that tannins inhibit parasitic infection (28). The reduction in FEC is due to tannins reducing GIN fecundity and inhibiting key enzymes, thereby inhibiting larval development (29–31), larval exsheathment (32), larval motility and migration (33), egg hatching (33), larval feeding (31), and motility of adults (26). In addition, the reduction of FEC is also attributed to the indirect effect of tannin (22). *H. contortus* is a blood-sucking parasite that feeds on the blood of pregnant ewes and can cause anemia and even death in pregnant ewes (34). *H. contortus* infection of pregnant ewes affects blood PCV levels (35). This is consistent with the findings of Castillo et al. (36), who reported that *H. contortus* significantly reduced PCV levels in sheep. Yet another way to determine the effect of *H. contortus* on pregnant ewes was by measuring their blood indicators (37). RBC is indicative of changes in erythrocyte levels after GIN infection (38, 39). Consistent with our study, infection with *H. contortus* resulted in decreased erythrocyte levels and increased EOS%, NEU%, MONO%, LY%, and PLT in pregnant ewes. Previous studies have found a negative correlation between lymphocytes and *H. contortus* fecundity (40). This can be explained by the fact that when the parasite infects the host, the host initiates lymphocytes to reduce *H. contortus* numbers and aggregates eosinophils to target and inhibit *H. contortus* development (41). Interestingly in this study, tannin increased PCV and RBC levels and decreased EOS%, NEU%, MONO%, LY%, and PLT in pregnant ewes infected with *H. contortus* (42). This may be primarily due to the reduction in FEC, and tannin enhanced host immunity, increased the absorption levels of amino acids and proteins to fight nematode infection, and restored blood loss (43).

It is well known that the hypothalamic–pituitary–gonadal axis (HPG) plays an important role in the reproduction of female animals, and the hypothalamus induces FSH and LH synthesis by releasing GnRH, which promotes the secretion of E_2_ and P_4_ (44). Hormones act through specific receptors, for example, FSH binds to the FSH receptor (FSH-R) and regulates E_2_ production (45, 46). However, the parasite can interfere with the synthesis, secretion, metabolism, and function of endogenous hormones in female animals and also interfere with the immune function of female animals (5). Previous studies have found the parasite can cause a decrease in the levels of FSH, LH, E_2_, and P_4_ in female animals (7, 14, 47). In this study, it was found that the levels of reproductive hormones (GnRH, E_2_, P_4_, hCG, FSH, and LH) and gonadal axis receptor mRNA were significantly decreased in pregnant ewes infected with *H. contortus*. It was previously reported that the lower LH and FSH levels of pregnant ewes in the GINs group may be due to the inhibition of GnRH (48, 49). At the same time, hCG maintains pregnancy by promoting luteinization and stimulating P_4_ production (50). Low levels of LH and hCG may lead to reduced activity of its receptors, resulting in lower levels of E_2_ and P_4_ (51)_._ In addition, tannin can promote the synthesis and release of hormones and inhibit the reproduction of GINs (18, 47).

There is growing evidence that there is a bidirectional relationship between the HPG and the immune system (52). Pregnancy hormones have a very important regulatory role in the maternal immune system (50). Parasitic infections during pregnancy make hormonal imbalances, leading to a disorder between immunity and inflammation (53). Bohstam et al. (54) suggested that the balance between pro-inflammatory factors, such as interleukin-1*β* (IL-1β), IL-2, IL-6, TNF-*α*, interferon-*γ* (IFN-γ), and IL-8, and anti-inflammatory factors, including transforming growth factor-β (TGF-β), IL-4, and IL-10, was a key factor in maintaining immune system homeostasis. Previous studies had found that parasitic infections led to an increase in inflammatory factors (IL-3 and IL-6) in sheep (55, 56). The results of this study showed that the levels of pro-inflammatory factors (IL-2, TNF-*α*, and IFN-*γ*) were increased and the levels of anti-inflammatory factors (TGF-*β*, IL-4, and IL-10) were decreased in pregnant ewes infected with *H. contortus*. In addition, we reported an increase in the mRNA expression level of TNF-α in the placenta. This was attributed to the activation of the inflammatory cascade induced by parasitic infection, and TNF-α stimulated the production of inflammatory cytokines (IL-3, IL-6, and IL-10) (57).

The placenta plays an important role in the development of the fetus by connecting the fetus to the mother, while all nutrients and waste products must pass through the placental barrier (58, 59). In addition, some parasites utilize this barrier to enter the fetal bloodstream (60). It is well known that the maintenance of a healthy pregnancy is associated with the secretion of TH1 cytokines (IFN-γ, IL-2, IL-12, and TNF-α) and TH2 cytokines (IL-4, IL-5, IL-6, IL-10, and IL-13) in the placenta (61, 62). However, parasitic infections disrupted the inflammatory balance within the placenta, leading to increased levels of IFN-γ and TNF-α, which adversely affected pregnancy (63). Similarly, the results of the present study showed that the levels of pro-inflammatory factors (IFN-γ, IL-2, and TNF-α) mRNA were elevated and the levels of inflammatory factors (IL-4, IL-10, and TGF-β) mRNA were decreased in the placenta and that tannins restored their levels.

Several studies have shown that parasitic infection of pregnant ewes can lead to fetal infection and abortion (13). To further investigate the effect of *H. contortus* on fetal development, we analyzed the fetal ovarian transcriptome through KEGG enrichment. The results indicated that KEGG pathways primarily localized genes to TH1 and TH2 cell differentiation, as well as to TH17 cell differentiation signaling pathways, in addition to gap junction, estrogen, and FOXO1 signaling pathways. As expected, infection during pregnancy triggers a TH1-type response in the placenta that affects fetal development (64). It has been found that the fetal immune system does not need to be fully mature to respond to parasitic infections. Among the immune cells involved, TH17 cells are the most abundant CD4+ T cells in mucosal tissues, secreting interleukin-17 (IL-17) and interleukin-22 (IL-22) to mediate immune responses (65). The results of the present study suggested that KEGG enrichment in the estrogen signaling pathway within the fetal ovary after infection may be due to systemic damage caused by the parasite affecting the host organism, as previously hypothesized (66). In addition, parasitic infections affect the synthesis of animal hormones (67). The results of the present study suggest that KEGG enrichment in the estrogen signaling pathway in the post-infected fetal ovary may be due to damage caused by the parasite (68), a hypothesis that has been proposed.

Tannins are plant secondary polyphenols, which are diverse and have a high affinity for proteins (69). As a result, tannins play an indirect role in boosting immunity by improving the protein availability of the host (70). Our results showed that after tannin was added to the diet, the levels of pro-inflammatory and anti-inflammatory factors were restored. This could be attributed to the fact that tannins, on the one hand, exert a repellent effect, reducing inflammation in pregnant ewes (71). On the other hand, polyphenols such as tannins have been shown to decrease the levels of several pro-inflammatory cytokines, including IL-6, IL-8, and TNF-*α* (72). Additionally, numerous studies have demonstrated that tannins can promote follicular development (47), increase E_2_ levels (73), and function as ovarian antioxidants (74). Therefore, the sequencing results clearly indicate that tannins restore ovarian function and may improve fetal growth and development. However, the exact mechanism requires further investigation.

## 5 Conclusion

In conclusion, our results demonstrated that *H. contortus* infection resulted in disturbance in serum reproductive hormones and inhibition of receptor mRNA levels, increased placental inflammation, and affected the fetal development. Surprisingly, tannin alleviates placental inflammation and fetal growth impairment in pregnant ewes caused by *H. contortus* infection. According to the European Food Safety Authority (EFSA) evaluations, tannins have potential benefits as feed additives at certain concentrations, especially in terms of antiparasitic and improved intestinal health (75). Thus, tannin may be a new additive to improve the reproduction ability of sheep infected with *H. contortus*. The results provided fundamental knowledge for a healthy feeding strategy for sheep breeding.

## Data Availability

The datasets presented in this study can be found in online repositories. The names of the repository/repositories and accession number(s) can be found in the article/supplementary material.

## Footnotes

^1^ http://daehwankimlab.github.io/hisat2/
